# Impact of Stokes Shift on the Performance of Near-Infrared Harvesting Transparent Luminescent Solar Concentrators

**DOI:** 10.1038/s41598-018-34442-3

**Published:** 2018-11-05

**Authors:** Chenchen Yang, Jun Zhang, Wei-Tao Peng, Wei Sheng, Dianyi Liu, Padmanaban S. Kuttipillai, Margaret Young, Matthew R. Donahue, Benjamin G. Levine, Babak Borhan, Richard R. Lunt

**Affiliations:** 1 Department of Chemical Engineering and Materials Science, Michigan State University, East Lansing, MI 48824 USA; 2 Department of Chemistry, Michigan State University, East Lansing, MI 48824 USA; 3 Department of Physics and Astronomy, Michigan State University, East Lansing, MI 48824 USA

## Abstract

Visibly transparent luminescent solar concentrators (TLSC) have the potential to turn existing infrastructures into net-zero-energy buildings. However, the reabsorption loss currently limits the device performance and scalability. This loss is typically defined by the Stokes shift between the absorption and emission spectra of luminophores. In this work, the Stokes shifts (*SS*) of near-infrared selective-harvesting cyanines are altered by substitution of the central methine carbon with dialkylamines. We demonstrate varying *SS* with values over 80 nm and ideal infrared-visible absorption cutoffs. The corresponding TLSC with such modification shows a power conversion efficiency (*PCE*) of 0.4% for a >25 cm^2^ device area with excellent visible transparency >80% and up to 0.6% PCE over smaller areas. However, experiments and simulations show that it is not the Stokes shift that is critical, but the total degree of overlap that depends on the shape of the absorption tails. We show with a series of *SS*-modulated cyanine dyes that the *SS* is not necessarily correlated to improvements in performance or scalability. Accordingly, we define a new parameter, the overlap integral, to sensitively correlate reabsorption losses in any LSC. In deriving this parameter, new approaches to improve the scalability and performance are discussed to fully optimize TLSC designs to enhance commercialization efforts.

## Introduction

Seamless installation of transparent luminescent solar concentrator systems onto the architectural envelope transforms passive surfaces into solar energy harvesting resources, dramatically improving energy utilization efficiency without compromising their current functionality and aesthetic quality underneath^1–3^. However, reabsorption of emitted photons combined with non-unity quantum yield (*QY*) of the luminophores act as the main loss mechanism that can dramatically limit the power conversion efficiency over larger areas (transparent, colored, or opaque). Reabsorption loss originates from the overlap between the absorption and emission spectra^4,5^. The Stokes shift (*SS*), defined as the wavelength difference between the absorption and emission peak maxima for the same transition, is frequently used to quantify and rationalize this loss in LSCs.

Recently, several approaches towards mitigating the reabsorption effect by increasing the Stokes shift with various species of luminophores have been reported for quantum dots^2,6–15^, rare-earth ions^16–23^, nanoclusters^2,12,24,25^, and organic molecules^26–31^. For example, inorganic semiconductor nanocrystals exhibit high photoluminescence efficiencies with absorption and emission spectra that are tunable by particle size and composition. Several strategies have been developed to increase the Stokes shift, including most notably the formation of core/shell “giant” quantum dots (QDs) as quasi-type I or type II hetero-structures for CdSe/CdS^7,12^, PbS/CdS^14^ or I-II-VI_2_ ternary CdSe/Cd_x_Pb_1-x_S^8^, CuInS_2_/CdS^9^, CuInS_2_/ZnS QDs^11^. The nanocrystal shell typically has a larger energy bandgap, acting as a photon absorbing antenna and protective carrier barrier when energy is transferred to the lower bandgap core crystal photon emitter. The energy gap difference between the core and the shell crystals results in an increase in downshift up to 150–200 nm^8,14^. Doping quantum dots with transition metal ions is another approach to tackle the reabsorption problem, for example, utilizing Mn-doped ZnSe^6,14^, Cu-doped CdSe QDs or nanoplatelets^15^. The doping impurity introduces new localized excited energy states (mid-gaps) within the original QD energy bandgap, which generates a downshifted radiative recombination with respect to the absorption.

However, a key limitation of these QDs is the continuous band-like absorption profiles that hinder selective absorption of invisible infrared photons without an accompanying absorption in the visible that reduce their transparency and aesthetic quality. In contrast, organic molecules are a class of luminophore candidates for LSC and TLSC applications that exhibit excitonic properties and separated molecular orbitals stemming from their π-conjugated molecular structure. While the Stokes shifts of traditional and commercially available organic dyes utilized in LSCs are generally small (<20–30 nm)^26,29,30,32^, recent efforts have looked to circumvent the reabsorption loss by using an excitation energy transfer (energy migration) strategy with multiple dyes via Förster resonance energy transfer (FRET)^33–37^. Such an approach separates the absorption of the donor from the emission of the acceptor so that the reabsorption in the LSCs is reduced but the close physical coupling of the dyes along with the need for multiple dyes with high quantum yields creates additional challenges. Another method has also been explored via resonance shifting in optical cavity designs for the waveguides^38^, but it requires the utilization of neat thin-film layers of luminophores which are often less suitable for achieving the highest luminescent quantum yields.

For many TLSC applications, high aesthetic quality and transparency are the most critical metrics^39^. Thus, harvesting the invisible portion of the solar spectrum (ultraviolet (UV) and near-infrared (NIR)) is most beneficial for such applications^4^. TLSC with NIR selective harvesting cyanine dyes has been demonstrated in previous work but the Stokes shifts were all <30 nm, thus limiting the larger area optimization^3^. In this work, we develop large Stokes shift TLSCs by modifying the central methine coordination of NIR selective cyanine dyes, resulting in Stokes shifts over 80 nm with simultaneously improved *QY* and maintaining selective NIR harvesting. These changes in *SS* are explained by ab initio calculations which show that the distortion about the central amino group in the excited states decreases the energy of the lowest unoccupied molecular orbitals (LUMO) energy. The corresponding TLSC devices exhibit a 30% *PCE* improvement for a 25.8 cm^2^ device that was found to stem, surprisingly, not from the changes in *SS* but from changes in the absorption width and improved quantum yield. These trends are quantitatively confirmed by distance dependence quantum efficiency measurements and optical modeling. Thus, we introduce a new parameter to replace the Stokes shift, the overlap integral, to more accurately correlate the true reabsorption loss, act as a fast screening parameter, and prevent misleading expectations in performance. In deriving this parameter, new approaches towards minimizing the loss to reabsorption are proposed as a roadmap for both LSC and TLSC designs to help realize their full potential^4^.

**Figure 1:**
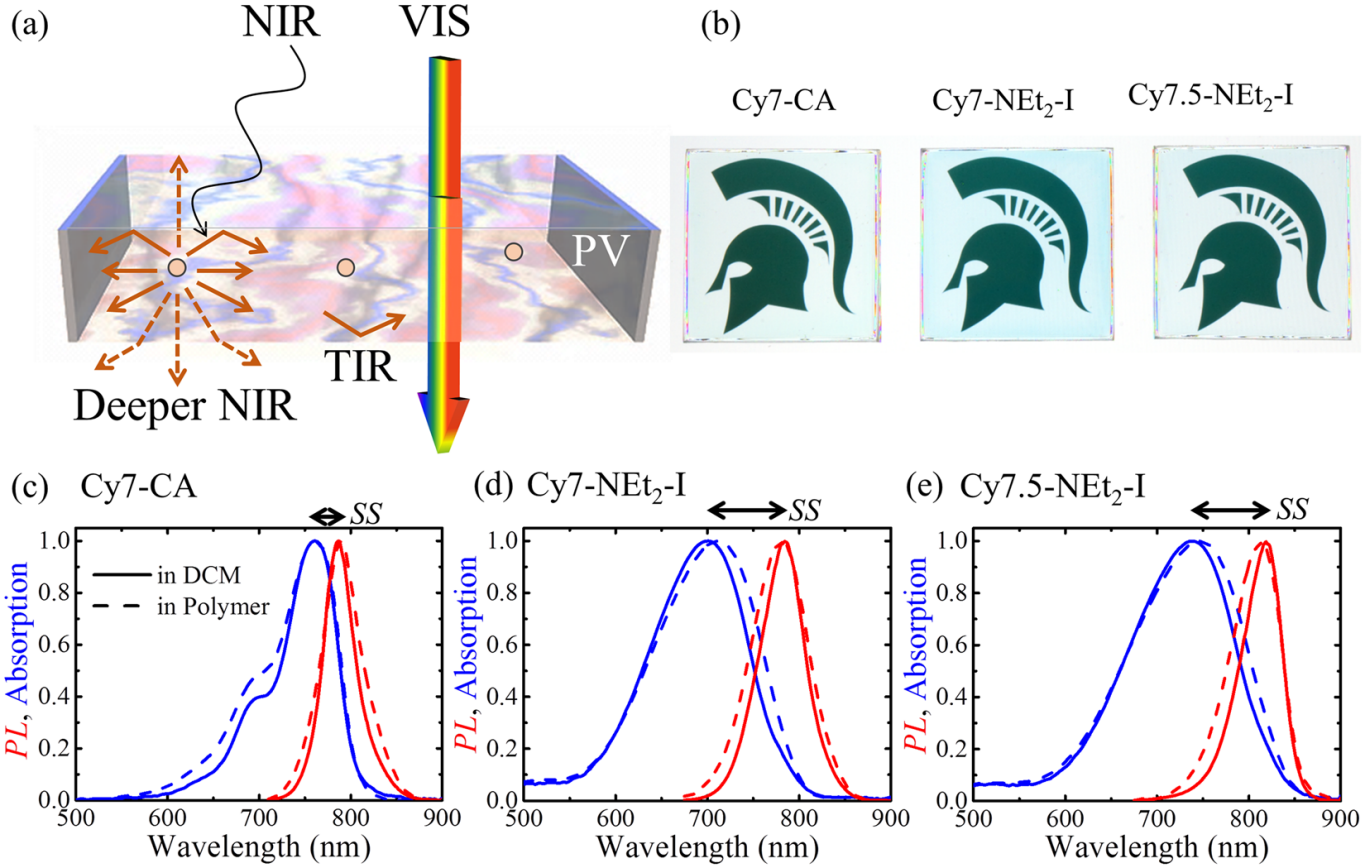
(**a**) Schematic showing a transparent luminescent solar concentrator (LSC) that selectively harvests near-infrared (NIR) light and emits deeper NIR while passing visible light. (**b**) Photographs of the TLSCs in front of the MSU Spartan helmet incorporating Cy7-CA, Cy7-NEt_2_-I and Cy7.5-NEt_2_-I luminophores (illuminated from behind the TLSC). Normalized absorption (blue) and emission spectra (red) of Cy7-CA (**c**) Cy7-NEt_2_-I (**d**) and Cy7.5-NEt_2_-I (**e**) in DCM solutions (solid lines) and polymer films (dashed lines). Permission to utilize the Spartan helmet logo is kindly provided by MSU.

**Figure 2:**
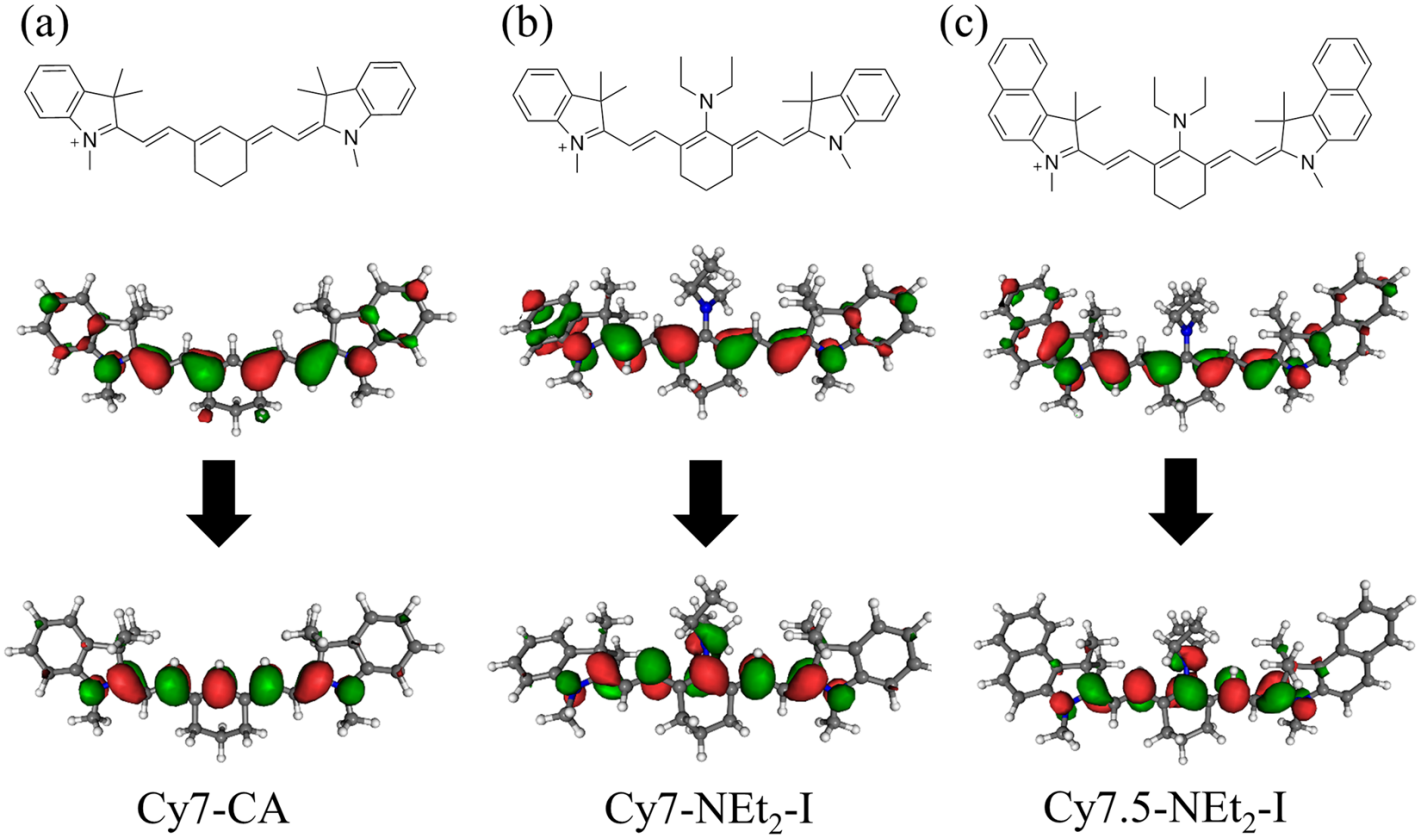
Molecular structure, HOMO and LUMO electronic orbitals of Cy7-CA (**a**) Cy7-NEt_2_-I (**b**) and Cy7.5-NEt_2_-I (**c**).

We focus on two key parent cyanine salts that are derivatized to modify the Stokes shift: 2-((*E*)-2-((*E*)-2-chloro-3-(2-((*E*)-1,3,3-trimethylindolin-2-ylidene)ethylidene)cyclohex-1-en-1-yl)vinyl)-1,3,3-trimethyl-3*H*-indol-1-ium iodide and 2-((*E*)-2-((*E*)-2-chloro-3-((*E*)-2-(1,1,3-trimethyl-1,3-dihydro-2*H*-benzo[e]indol-2-ylidene)ethylidene)cyclohex-1-en-1-yl)vinyl)-1,1,3-trimethyl-1*H*-benzo[e]indol-3-ium iodide. These parent compounds are converted via the addition/elimination reaction of the Cl on the central methine backbone to 2-((*E*)-2-((*E*)-2-(diethylamino)-3-(2-((*E*)-1,3,3-trimethylindolin-2-ylidene)ethylidene)cyclohex-1-en-1-yl)vinyl)-1,3,3-trimethyl-3*H*-indol-1-ium iodide (Cy7-NEt_2_-I) and 2-((*E*)-2-((*E*)-2-(diethylamino)-3-((*E*)-2-(3-ethyl-1,1-dimethyl-1,3-dihydro-2*H*-benzo[*e*]indol-2-ylidene)ethylidene)cyclohex-1-en-1-yl)vinyl)-3-ethyl-1,1-dimethyl-1*H*-benzo[*e*]indol-3-ium iodide (Cy7.5-NEt_2_-I). In addition, 1-(5-carboxypentyl)-3,3-dimethyl-2-((*E*)-2-((*E*)-3((*E*)-2-(1,3,3-trimethylindolin-2ylidene)ethylidene)cyclohex-1-enyl)vinyl)-3*H*-indolium chloride (Cy7-CA) introduced previously is also included for comparison. We have tested a large number of different substituents, substituted at C4^40^ with *SS* ranging from <20 nm to >180 nm. The result of those studies will be described in more detail elsewhere; in this work, we focus on these two particular derivatives for device integration as they provided the highest *SS* and *QY* with selective absorption in NIR range of the solar spectrum. The absorption and emission spectra in both dichloromethane (DCM) solution and in a polymer matrix of Cy7-CA, Cy7-NEt2-I and Cy7.5-NEt2-I are plotted in Fig. 1c–e. The truncated molecular structures of all three cyanine dyes are shown in Fig. 2. Cy7-CA acts as the control luminophore with a small Stoke shift of 27 nm in DCM. The Stokes shift of the diethylamino substituted analog, Cy7-NEt_2_-I is increased to 84 nm with a *QY* of 30 ± 2% and absorption peak of 700 nm in DCM. Similarly, the modified Cy7.5-NEt_2_-I also shows an increased *SS* of 81 nm with red-shifted absorption peak of 738 nm compared to Cy7-NEt_2_-I. The *QY*s of these three cyanine dyes are summarized in Table 1 where the *QY* of the three cyanine dyes in the polymer film are modestly reduced compared to that in DCM solution. Density functional theory based calculations shown in Fig. 2 are utilized to understand the mechanism of the Stokes shift variation. The NEt_2_ substitution leads to additional relaxation in the central amino groups of Cy7-NEt_2_-I and Cy7.5-NEt_2_-I, which is presumably responsible for the increased Stokes shift. We note that the spectral shifts moving from the solvent to the polymer matrix are quite small (only several nm) and are expected due to the combination of solvochromatic shifts and changes in steric hindrance. When the cyanine dyes are introduced into the polymers, the more polarized environment stabilized the excited state, leading to a bathochromic shift of the absorption. However, after the dye are mounted into the polymers, the free rotation of the dyes is highly limited, creating a vibration energy change at the ground state, thus leading to a hypsochromic shift of the *PL*.

**Table 1:** Summary of the absorption λ_max, emission λ_max, Stokes shifts (SS) and quantum yields (QYs) of Cy7-CA, Cy7-NEt2-I and Cy7.5-NEt2-I in DCM and polymer films.

		Absorption ${\lambda }_{max}$ (nm)	Emission ${\lambda }_{max}$ (nm)	*SS* (nm)	*QY* (%)
Cy7-CA	Solution	760 (1.631 eV)	787 (1.575 eV)	27	24 ± 1
	Polymer	762 (1.627 eV)	788 (1.573 eV)	26	19 ± 1
Cy7-NEt_2_-I	Solution	700 (1.771 eV)	784 (1.581 eV)	84	30 ± 2
	Polymer	710 (1.746 eV)	780 (1.590 eV)	70	26 ± 1
Cy7.5-NEt_2_-I	Solution	738 (1.680 eV)	819 (1.514 eV)	81	23 ± 1
	Polymer	746 (1.662 eV)	816 (1.519 eV)	70	15 ± 1

**Figure 3:**
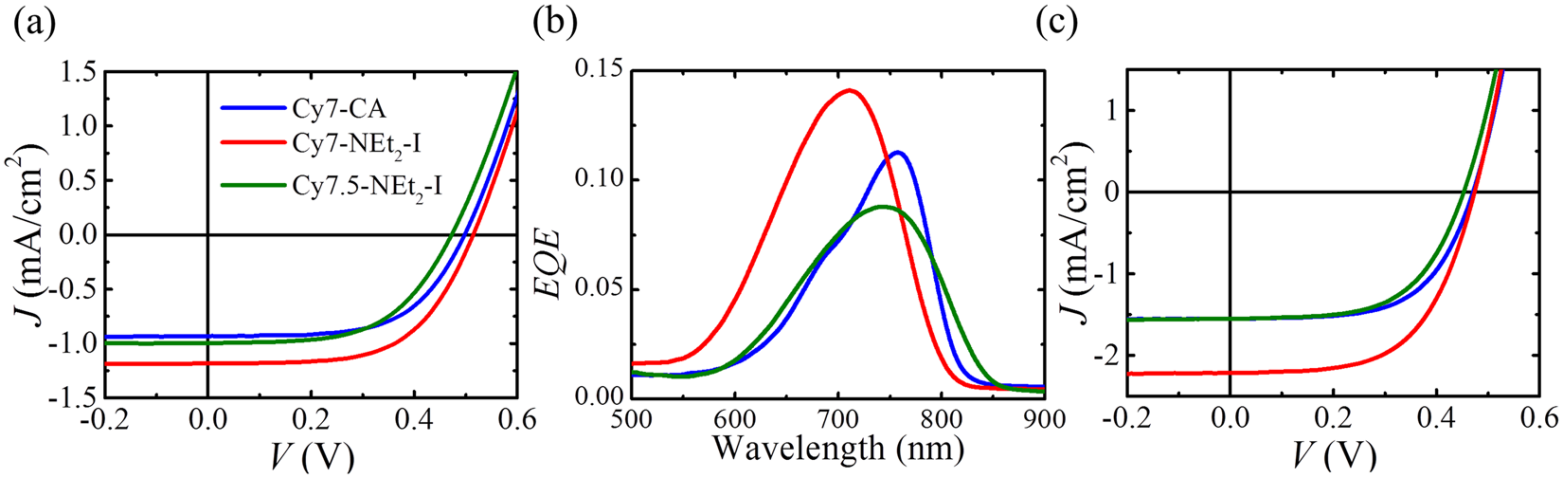
Current density as a function of voltage (*J-V* curves) for the fully assembled TLSC systems with three of the cyanine dyes based on waveguide dimension of 5.08 cm × 5.08 cm. (**b**) Representative external quantum efficiency (*EQE*) of three cyanine dye TLSC systems as a function of wavelength (measured at distance *d* = 5 mm). (**c**) *J-V* curves for the fully assembled TLSC systems with three of the cyanine dyes based on waveguide dimension 2.54 cm × 2.54 cm.

The TLSC devices are formed on borosilicate glass plates with an active area of 25.8 cm^2^. Cyanine molecules are dissolved in ethanol solutions, mixed with a polymer host, and then drop-cast onto glass sheets to form luminophore/polymer composite films. The polymer mounting medium acts to separate the dye molecules and prevent aggregation-induced quenching that reduces the *QY*. If the solvent or polymer mounting medium (or the combination of both) are poorly chosen, large clusters of the dye molecules will form which leads to visible nonuniformities. Moreover, if the solvent and polymer poorly paired, the mounting medium can separate from the solvent, resulting in wavy films with visible ripples that detrimentally affect the device aesthetic quality. Laser-cut Si photovoltaic cells are mounted around the two orthogonal edges and connected in parallel (see Experimental Section for details). The photovoltaic performance of the TLSCs based on the three cyanine dyes is shown in Fig. 3. The measured short-circuit current density (${J}_{SC}$) of the device with Cy7-NEt_2_-I is 1.18 mAcm^−2^, with an open-circuit voltage (${V}_{OC}$) of 0.51 V and a fill factor (*FF%*) of 60% leading to an efficiency of 0.36%. The *J-V* characteristic of TLSC with Cy7-CA shows a ${J}_{SC}$ of 0.93 mAcm^−2^, with a ${V}_{OC}$ of 0.49 V and *FF%* of 61%, resulting in a *PCE* of 0.28%, which is lower than previously reported Cy7 devices due to the 6-times larger device area. The second cyanine luminophore derivative Cy7.5-NEt_2_-I has similar ${V}_{OC}$ of 0.48 V and *FF%* of 57% with lower ${J}_{SC}$ of 1.02 mAcm^−2^, and an overall efficiency of 0.28% which is similar to the Cy7-CA control TLSC device. Figure 3b shows the external quantum efficiency (*EQE*) spectra of the three cyanine luminophores (see Experimental Section for details of the measurements). In general, the absorption spectra of the luminophores determine where the *EQE* peaks will be. This can be calculated as:

1 $$EQ{E}_{LSC}(\lambda )={\eta }_{opt}(\lambda )\frac{{\int }^{}EQ{E}_{PV}({\lambda }^{\text{'}})PL({\lambda }^{\text{'}})d{\lambda }^{\text{'}}}{{\int }^{}PL({\lambda }^{\text{'}})d{\lambda }^{\text{'}}}$$

where $EQ{E}_{LSC}(\lambda )$ is the *EQE* spectrum of the LSC system, which is the product of the LSC optical efficiency ${\eta }_{opt}(\lambda )$ at the absorption wavelength of the luminophore and the *EQE* of the edge-mounted PV cell over the emission wavelengths of the luminophore. To avoid confusion, it should be noted that the integrals here are performed over the wavelength range of the *PL* emission (${\lambda }^{\text{'}}$) not the wavelength of the incident light ($\lambda$). The peak positions of Cy7-CA, Cy7-NEt_2_-I and Cy7.5-NEt_2_-I match the absorption spectra in Fig. 1c–e and no direct excitation of the edge mounted solar cells is observed in the spectra. In the emission wavelength range of these three cyanine dyes (700 to 850 nm) the edge-mounted Si PV show a nearly constant *EQE* (~90%) so that Eq. 1 simplifies to $EQ{E}_{LSC}(\lambda )={\eta }_{opt}(\lambda )\cdot EQ{E}_{PV}$ where $EQ{E}_{PV}\cong 0.90$. While Cy7-NEt_2_-I with the highest *QY* in the polymer film leads to the highest *EQE* peak of 14.1% at 710 nm, the *EQE* peaks of Cy7-CA and Cy7.5-NEt_2_-I are 11.3% and 8.8% at 760 nm and 745 nm, respectively. These are also consistent with the *QY* trend of the three cyanine dyes. The integrated short circuit current density from integrating the product of the position-averaged *EQE* and the AM1.5 G solar spectrum is used to confirm the photocurrent density of the whole TLSC device. For TLSCs with 5.08 cm $\times$ 5.08 cm active area, five *EQE* spectra were tested as a function of the distance (*d*) from the excitation source to edge-mounted PV cell. Based on its wide absorption and high *QY* Cy7-NEt_2_-I yields an integrated $\,{J}_{SC}$ of 1.22 mAcm^−1^. The wider absorption peak of Cy7.5-NEt_2_-I compensates the slightly lower *QY* in the polymer film, thus exhibiting a close integrated $\,{J}_{SC}$ compared with Cy7-CA. All the integrated $\,{J}_{SC}$ values are within error of the photocurrent densities from *J-V* measurement. To check the validity of photon balance from the *EQE*, transmission (*T(λ))*, and reflection (*R(λ)*) spectra measurements of these devices, we show that $EQE(\lambda )+R(\lambda )+T(\lambda )\le 1$ is satisfied at each wavelength^39^. This validity check is plotted in Fig. S3 for each device. It is also worth noting that the TLSC devices with these three cyanine dyes have been made with a smaller active area (6.45 cm^2^) similar to prior work utilizing 1-(6-(2,5-dioxopyrrolidin-1-yloxy)-6-oxohexyl)-3,3-dimethyl-2-((*E*)-2-((*E*)-3-((*E*)-2-(1,3,3 trimethylindolin-2-ylidene)ethylidene)cyclohex-1-enyl)vinyl)-3*H*-indolium chloride (Cy7-NHS) that had an active area of 4 cm^2^ (Fig. 3 (c)). With similar device active area, the smaller TLSC with Cy7-NEt_2_-I exhibit significantly improved *PCE* of 0.62% (2.21 mAcm^−1^ of $\,{J}_{SC}$, 0.47 V of ${V}_{OC}$ and 60% of *FF%*) over previous work, while the TLSCs with the two other cyanine dyes, Cy7-CA and Cy7.5-NEt_2_-I, have very similar photovoltaic performance (*PCE*~0.4%) compared to previous work (Table 2)^3^.

**Table 2:** Summary of photovoltaic parameters and overlap parameters (J, S, S′, and OI) of TLSC systems with the different cyanine luminophores and Cy7-NHS (from ref ^3^). We note that only the OI accurately correlates to the scaling behavior measured and shown in Fig. 4.

	*Area* (cm^2^)	*Jsc* (mAcm^−2^)	*Int. Jsc* (mAcm^−2^)	*Voc* (V)	*FF* (%)	*PCE* (%)	*AVT* (%)	*CRI*	*J* (μm^3^M^−1^)	*S*	*S*′	*OI*
Cy7-NHS ^3^	4	1.2 ± 0.1	1.00	0.50 ± 0.01	66 ± 2	0.40 ± 0.03	87.7	91.0	1.49	4.07	1.84	27.9
Cy7-CA	6.45	1.55 ± 0.05	0.96	0.47 ± 0.01	61 ± 1	0.44 ± 0.02	88.1	92.1	1.56	3.49	1.72	27.2
	25.8	0.93 ± 0.02		0.49 ± 0.01	61 ± 1	0.28 ± 0.02						
Cy7-NEt_2_-I	6.45	2.2 ± 0.2	1.22	0.47 ± 0.01	60 ± 1	0.62 ± 0.05	77.1	75.6	0.46	10.22	3.42	25.9
	25.8	1.18 ± 0.01		0.51 ± 0.01	60 ± 1	0.36 ± 0.01						
Cy7.5-NEt_2_-I	6.45	1.55 ± 0.09	0.82	0.46 ± 0.01	59 ± 1	0.41 ± 0.03	84.7	89.4	0.99	7.22	2.82	30.8
	25.8	1.02 ± 0.01		0.48 ± 0.01	57 ± 1	0.28 ± 0.02						

**Figure 4:**
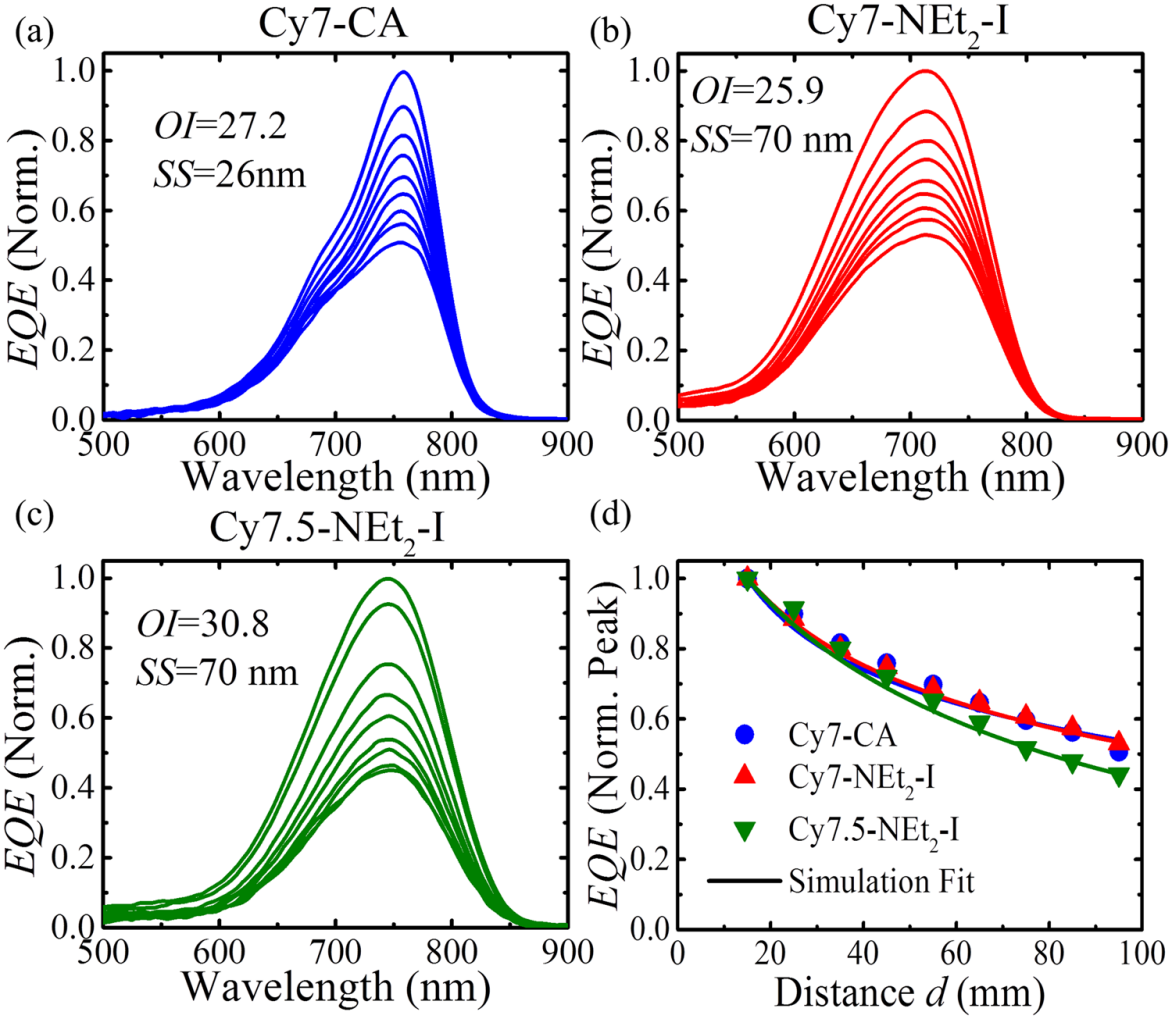
Position-dependent *EQE* of Cy7-CA (**a**), Cy7-NEt_2_-I (**b**) and Cy7.5-NEt_2_-I (**c**) as a function of wavelength measured from 15 mm to 95 mm, with 10 mm increments. (**d**) Calculated optical efficiencies (solid lines) as a function of distance *d* of three cyanine luminophore TLSC systems to fit the measured normalized *EQE* peak values (symbols).

To explore the impact of Stokes shift on the scalability, TLSC systems were characterized by the external quantum efficiency as a function of position. Multiple *EQE* scans were taken for each TLSC system as *d* was increased from 15 mm to 95 mm (10 mm interval, and the same Si PV strip was used for all the *EQE* scans). The normalized *EQE* spectra of three cyanine dyes were plotted in Fig. 4a–c. The *EQE* peak values of each individual scan of the three cyanine dyes were extracted and plotted in Fig. 4d.

The overall power conversion efficiency of an LSC or TLSC system is the product of the component efficiencies^1,4^:

2 $${\eta }_{LSC}={\eta }_{Opt}^{\ast }\cdot {\eta }_{PV}^{\ast }=(1-{R}_{f})\cdot {\eta }_{Abs}\cdot {\eta }_{PL}\cdot {\eta }_{Trap}\cdot {\eta }_{RA}\cdot {\eta }_{PV}^{\ast }$$

where ${R}_{f}$ is the front surface reflection, ${\eta }_{Abs}$ is the solar spectrum absorption efficiency of the luminophore, ${\eta }_{PL}$ is the luminescence efficiency of the luminophore, ${\eta }_{Trap}$ is the photon trapping (or waveguiding) efficiency, ${\eta }_{PV}^{\ast }$ is the efficiency of the edge-mounted PV cell normalized by its solar spectrum absorption efficiency and *EQE* at the luminophore wavelength to account for photon downshifting, and ${\eta }_{RA}$ is the efficiency of suppressing reabsorption^4,5,41^ (see Supporting Information for details). We reemphasize that ${\eta }_{Opt}^{\ast }=(1-{R}_{f})\cdot {\eta }_{Abs}\cdot {\eta }_{PL}\cdot {\eta }_{Trap}\cdot {\eta }_{RA}$ is the overall optical efficiency, which is defined as the ratio of number of photons reaching the waveguide edge to the number of photons incident on the waveguide front active surface across all incident wavelengths. In Eq. 1, ${\eta }_{opt}(\lambda )\,\,$is the optical efficiency at a specific wavelength $(\lambda )$ of the incident light utilized for quantum efficiency calculations so that ${\eta }_{opt}(\lambda )=(1-{R}_{f})\cdot A(\lambda )\cdot {\eta }_{PL}\cdot {\eta }_{Trap}\cdot {\eta }_{RA}$. Here, $A(\lambda )$ is the normal incidence absolute absorption spectrum. It is assumed that all the other loss factors $\,({\eta }_{PL}$, ${R}_{f}$, ${\eta }_{Trap}$ and ${\eta }_{RA}$) are essentially independent of wavelength. Both optical efficiencies (${\eta }_{Opt}^{\ast }$ and ${\eta }_{opt}(\lambda )$) have similar roll-off behavior as a function of distance (*d*), since both are predominantly limited by reabsorption loss.

The higher degree of conjugation with Cy7.5-NEt_2_-I results in a lower bandgap. Therefore, both the absorption and emission spectra of Cy7.5-NEt_2_-I are red-shifted compared to Cy7-NEt_2_-I, and the shape of both spectra remain nearly identical. That the absorption spectrum of Cy7-NEt_2_-I is blue-shifted relative to Cy7-CA can be understood via the Dewar-Knott color rule^42–44^. Addition of an electron donating species to the central position of the polymethine chain destabilizes the LUMO, which has significant density on the central carbon (Fig. 2), while leaving the energy of the HOMO, which has little density on that carbon, unaffected. When the amine is oriented such that its lone pair electron is conjugated with the polymethine chain, it acts as such an electron donating group, therefore the excitation energy is increased.

The nuclear relaxation responsible for the enhanced Stokes shift of Cy7-NEt_2_-I and Cy7.5-NEt_2_-I compared to Cy7-CA was investigated via linear response time-dependent density functional theory calculations. The computed Stokes shifts (0.07, 0.15, and 0.17 eV for truncated models of Cy7-CA, Cy7-NEt_2_-I, and Cy7.5-NEt_2_-I, respectively) are in good agreement with the experimental values (0.06, 0.19, and 0.17 eV in solution). The polymethine chains of all three molecules relax similarly in the excited state, with individual bond lengths changing by up to 0.02 Å. Additional relaxation is observed in the central amino groups of Cy7-NEt_2_-I and Cy7.5-NEt_2_-I. The bond lengths between the central carbon of the polymethine chain and the amine nitrogen are lengthened by 0.02 Å, consistent with excitation into a LUMO that is antibonding with respect to the amine bond (Fig. 2). The amino group also twists relative to the polymethine chain upon excitation, with the torsion angles increasing from 44° to 55° in Cy7-NEt_2_-I and from 44° to 56° in Cy7.5-NEt_2_-I. This torsional motion reduces the conjugation of the lone pair on the nitrogen atom of the amino group to the π orbitals of the polymethine chain, thus decreasing the electron donating ability of the group. Together, these twisting and stretching motions destabilize the LUMO but leave the energy of the HOMO—which does not have density on the amine bond—relatively unaffected. These changes in orbital energy account for the enhanced Stokes shifts of Cy7-NEt_2_-I and Cy7.5-NEt_2_-I compared to Cy7-CA.

The main factor that leads to enhanced performance for the TLSC with Cy7-NEt_2_-I is the increased ${J}_{SC}$ (Table 2). Several key factors are responsible for these changes: changes in quantum yield, variations in total absorption (absorption width), and reductions in reabsorption loss. Surprisingly, the Cy7-NEt_2_-I with a large Stokes shift of 70 nm in the polymer film only shows slightly better reabsorption efficiency compared to Cy7-CA with a small Stoke shift of 26 nm, and Cy7.5-NEt_2_-I shows even more rapid *EQE* peak decay than the other two. To understand this surprising result, we remember that the *EQE* of TLSC consists of the optical efficiency, ${\eta }_{Opt}$ (the number of photons reaching the waveguide/number of photons incident on the waveguide active area) and the *EQE* of the PV at the emission wavelength. A numerical calculation of optical efficiency was performed by accounting for multiple reabsorption and emission events (see Supporting Information). Excellent agreement of the experimental *EQE* and the simulated optical efficiency suggests that reabsorption is indeed the main loss mechanism in all of these TLSC systems and that despite the large increase in the *SS*, the scalability is not significantly improved. Considering the other factors contributing to the photocurrent, it is primarily the increased absorption width and quantum yield that leads to the ${J}_{SC}$ increase.

Both the experimental and modeling result suggests that Stokes shift is not ultimately a useful design parameter to identify how well a luminophore will perform in LSCs over large area. Here we define a new parameter, the overlap integral (*OI*) to quantify the reabsorption properties of a luminophore as:

3 $$OI={\int }_{0}^{\infty }A(\lambda )\cdot P{L}^{\ast }(\lambda )d\lambda $$

where *A(λ)* is the absolute absorption spectrum (calculated by $A(\lambda )=1-R(\lambda )-T(\lambda )$, $R(\lambda )$ is the reflection spectrum, and $T(\lambda )$ is the transmission spectrum) of a luminophore/host composite film, and PL^*^(λ) is the normalized emission spectrum of the luminophore in the host material. The *OI* then depends on the thickness of the luminophore layer and the degree of overlap between the absorption and emission spectra in the host material (rather than in solution). The calculated *OI* for the investigated luminophores is summarized in Table 2. Despite the large *SS* between Cy7-CA and Cy7-NEt_2_-I and between Cy7-CA and Cy7.5-NEt_2_-I, there is only a small difference in the *OI*. This is because the absorption tail is broadened with the increase in the *SS*. Peak broadening is often observed with red-shifted chromophores, resulting from increased vibrational states and/or larger conformational flexibility and more potential isomeric states. Furthermore, a potential charge transfer process can also lead to spectral broadening, if a strong dipole is induced upon excitation. The similarity of the *OI* elucidates the similar optical efficiency (or *EQE* decay curves) in Fig. 4d and show the correct correlation: decreasing the *OI* leads to improved scalability in the *EQE* while the measured *SS* shows an incorrect trend. Thus, the *OI* more sensitively captures and reflects the distance dependence of luminophores with differing Stokes shifts. The way the *OI* is defined in Eq. 3 it is useful not only for organic dyes but all luminophores (including inorganic quantum dots) and all luminophore optical densities. For screening purposes the *OI* can be evaluated for fixed absolute peak (or specified wavelength) absorption values (e.g. *A* = 80%). It is thus a useful design parameter for quickly tracking, predicting, and understanding relative performance changes in a range of LSC systems.

It is natural to consider the FRET overlap integral (*J*) as a parameter to correlate the *EQE* scalability as it is material specific. The well-known expression for *J* is:

4 $$J=\frac{{\int }^{}P{L}_{D}(\lambda ){{\epsilon }}_{A}(\lambda ){\lambda }^{4}d\lambda }{{\int }^{}P{L}_{D}(\lambda )d\lambda },$$

where $P{L}_{D}(\lambda )$ is the emission spectrum of the donor dye, and ${{\epsilon }}_{A}(\lambda )$ is the molar absorptivity coefficient of the acceptor dye. Conceptually, however, *J* depends on both the shape and the magnitude of molar absorptivity coefficient (${{\epsilon }}_{A}$) and therefore can predict the wrong scaling. For example, if the magnitude of ${{\epsilon }}_{A}$ is lower for the same spectral shape, one would simply load more luminophore to maintain the same optical density (absolute absorption) leading to the same *OI* but lower *J*. Indeed, we find that *J* does not correlate with the scaling of the *EQE* with plate length. The measured trends, from lowest to highest scaling are: Cy7.5-NEt_2_-I, Cy7-CA, Cy7-NEt_2_-I; *J* predicts Cy7-CA, Cy7.5-NEt_2_-I, Cy7-NEt_2_-I while the *OI* correctly captures the trend Cy7.5-NEt_2_-I, Cy7-CA, Cy7-NEt_2_-I. In addition to *J*, the parameter, *S*, has been defined previously to try to quantify the reabsorption loss behavior^36^. This parameter was defined as the ratio of the absorption coefficients at the absorption and emission maxima in solution. We also defined a variation of the self-absorption ratio *S*′, defined as the ratio of the absolute absorption values at the absorption and emission maxima that can be used for solid films where there are subtle but important shifts in spectra. We calculate both *S* and *S*’ for each luminophore (Table 2) and find not only do these parameters give opposing trends, neither accurately captures the observed scaling trend.

**Figure 5:**
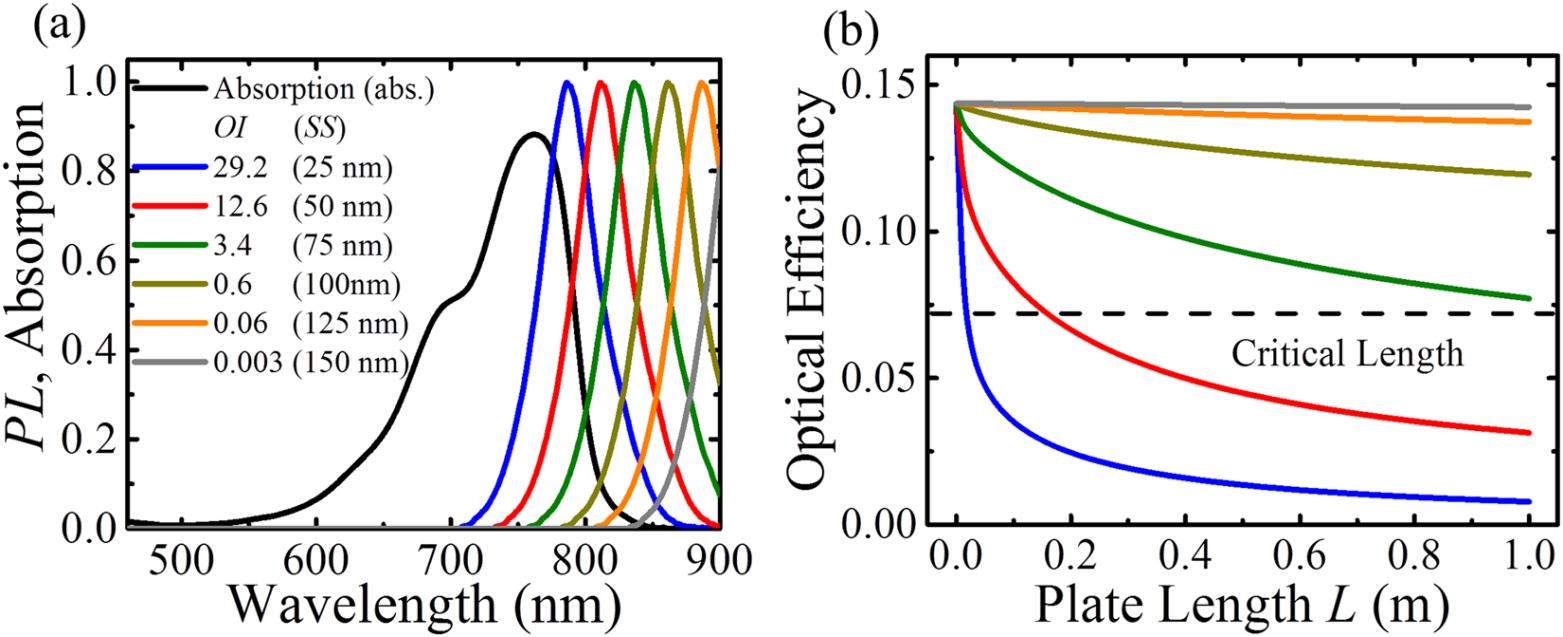
(**a**) Different overlap integral (*OI*) values obtained by keeping the absolute absorption fixed and shifting the normalized emission spectrum of a luminophore (Cy7-CA). We note that the *OI* and *SS* are not typically equivalent because chemical changes that lead to changes in the *SS* also lead to spectral changes in the tail absorption. The *SS* values for the simulation are provided to emphasize the reason that this parameter has been misleadingly considered as a design parameter. (**b**) Optical efficiencies as a function of plate length *L* for different *OI* values.

Given that the absorption coefficient in the NIR range for the host polymer material and targeted glass is in the range from 10^−2^ to 10^−4^ cm^−1^ (e.g. poly(methyl methacrylate)s and certain glasses) most reabsorption losses stems from the luminophore. To qualitatively connect the trends in the *OI* with the quantum efficiency roll-off behavior, we performed an additional simulation where the absolute absorption spectrum of a sample LSC was kept fixed and the normalized emission was manually shifted to create different overlap in the modeling as shown in Fig. 5a. We emphasize that while such a manual shifting of the emission corresponds to increases in the Stokes shift and creates different *OI* values, such an increase in the Stokes shift in practice does not necessarily lead to changes in the *OI* due to variations in the peak bandwidth. The calculated optical efficiency in Fig. 5b shows the impact of overlap integral on the TLSC scalability: if the *OI* can be decreased from 30 to 3 (i.e. an order of magnitude), the critical TLSC plate length (*L*) defined as the distance at which the optical efficiency decays to half of its original value can be increased from 2 cm to >1 m, which would be sufficient for many large-scale and window-based applications. We also note that there is a tradeoff between absorption efficiency and the *OI* on the total power conversion efficiency that also depends on the thickness (which dictates the optical density of the luminophore). In the case of the TLSCs, the impact on the average visible transparency (*AVT*) and color rendering index (*CRI*) should also be considered^39^, emphasizing that broad absorption in the invisible solar spectrum with sharp absorption cutoffs around the visible and near the bandgap are also key. Thus, strategies for sharpening the densities of states around the bandgap should be explored moving forward. The most feasible approach to minimize *OI* is to modify the NIR selective harvesting luminophore so that both the absorption edges have sharp cutoffs, keeping the emission narrow as well. Adding more fused rings to lock the geometry of the cyanine dyes could potentially restrict the distribution of conformers at the ground state, and thus achieve sharper transition bandwidths. Furthermore, the restricted geometry (molecular rigidity) could lead to increased quantum efficiency and luminophore lifetime by reducing non-radiative decay pathways. The lifetime of cyanine dyes is another interesting and key area. The fabricated TLSC devices show no degradation during >2 hours in exposure to air. Interestingly, we have shown that the stability of such compounds integrated as active layers in photovoltaic devices (a more demanding application than LSCs) are more a function of the coordinating counterion than the photoactive counterion itself. We have shown that such anion modifications can lead to photovoltaic lifetime anywhere as short as few days to >7 years for the same photoactive cation^45^. Thus, future efforts in characterizing the lifetime of these compounds in TLSC application will need to focus on both chemical modifications as well as ion pairing.

It is also important to consider the impact of increasing the *OI* by simply increasing the Stokes shift. The ideal Stokes shift should not only be engineered to minimize the *OI* but also carefully adjusted to allow all the emission of the luminophores to coincide with the high *EQE* wavelength range of the edge-mounted PV cells. If the *SS* is too large, a portion of the emission will have a wavelength longer than the absorption cut-off of the edge-mounted PVs and thus cannot be harvested. For example, with edge mounted Si PVs the maximum Stokes shift should be limited to <200 nm with the selective harvesting of a 200–300 nm slice of the NIR spectrum for optimal performance. For GaAs PVs with wider band gap the maximum Stokes shift is even more restricted.

In conclusion, we have synthesized novel NIR selective harvesting cyanine dyes with selective NIR harvesting, large Stokes shift >80 nm, and improved quantum yield of >30%. Luminescent solar concentrators based on these cyanine dyes exhibit power conversion efficiency of up to 0.6% combined with high visible transparency >80%. Based on the analysis of both experiment and simulation results, we show that the Stokes shift is not a suitable design parameter to quantify the reabsorption loss moving forward. Instead, we define a new parameter, the spectral “overlap integral”, derived in way to accurately capture the properties of the overlap between absorption and emission of a luminophore in future LSC design and optimization. Simulations also indicates that with one order of magnitude decrease of the overlap integral the LSC plate size can be increased to ~1 m, which is sufficient for most structural glazing systems. Thus, this work provides a guide to improve the efficiency and scalability of NIR-selective harvesting TLSC systems (and all LSCs) that can help fulfil the promise of low cost transparent solar cells.

## Experimental Section

### Module Fabrication

100 mgL^−1^ Cy7-CA (150 mgL^−1^ Cy7-NEt_2_-I or 150 mgL^−1^ Cy7.5-NEt_2_-I) ethanol solution was mixed with mounting medium (Fluoroshield F6182, Sigma-Aldrich) at a volume ratio of 1:2. This mixture was drop-cast on 5.08 cm $\times$ 5.08 cm $\times$ 0.635 cm glass sheets (for photovoltaic characterization) twice and allowed to dry for 6 h for each layer in a glove-box filled with nitrogen gas (O_2_, H_2_O < 1 ppm), resulting in a layer thickness of approximately 0.5 mm. Dichloromethane was mixed with (poly)-butyl methacrylate-co-methyl methacrylate (PBMMA) (Sigma-Aldrich) at a volume ratio of 1:1. This mixture was then drop cast onto the dye/Fluoroshield composite film to make a smooth and flat surface to avoid light scattering in the waveguide and act as a protection layer. The same layer structure was applied for 2.54 cm × 2.54 cm × 0.1 cm (for *PL* measurements) or 1.27 cm × 1.27 cm × 0.07 cm (for *QY* measurements). For photovoltaic measurements, single-crystalline solar cells (Vikocell) were laser cut into 2.54 cm × 0.635 cm and 5.08 cm × 0.635 cm strips for *PCE* and *EQE* measurements and 10.16 cm $\times$ 0.635 cm strips for *EQE* versus *SS* measurements. For *PCE* measurements, two PV strips were mounted on orthogonal edges using index matching gel (Thorlabs) to attach the PV strips on glass edges seamlessly and were connected in parallel. The remaining two edges were covered with specular film reflector (DF2000MA series, 3 M). For *EQE* measurements, one PV strip was attached to one edge of the waveguide with the other three edges painted black.

### Optical Characterization

Specular transmittance of both solutions and films were measured using a dual-beam Lambda 800 UV/VIS spectrometer in the transmission mode. The *PL* for the three cyanine dyes in both solutions and polymer films were measured by using a PTI QuantaMaster 40 spectrofluorometer with excitation at 675 nm for Cy7-CA, 650 nm for Cy7-NEt_2_-I and Cy7.5-NEt_2_-I. Quantum yield measurements were tested by using Hamamatsu Quantaurus fluorometer, excitation ranges in scan mode (10 nm per scan step) were adjusted to 700–750 nm for Cy7-CA, 650–700 nm for Cy7-NEt_2_-I and 680–730 nm for Cy7.5-NEt_2_-I. Six *QY* values were collected for each individual cyanine dye, and the reported *QY* for each cyanine dye in Table 1 was averaged from these six *QY* values with corresponding excitation wavelengths.

### Module Photovoltaic Characterization

*J-V* measurements were obtained using a Keithley 2420 source measurement under simulated AM1.5 G solar illumination (xenon arc lamp with the spectral-mismatch factor of 0.97 ± 0.03 for all the devices tested). The light intensity was calibrated with an NREL-calibrated Si reference cell with KG5 filter. For position-dependent *EQE* measurements, the excitation beam was obtained by directing chopped incident light from a quartz tungsten halogen lamp through a monochromator. *EQE* scans were performed by positioning the monochromatic excitation beam from a fiber perpendicular to the LSC waveguide front surface at various distances from a single edge-mounted Si PV cell. The measured *EQE* was corrected by the geometric factor, $g=\pi /ta{n}^{-1}(L/2d)$, which accounts for the different angle subtended by the solar cell at various distance *d*, where *L* is the LSC plate length. Note both *PCE* and *EQE* measurements for each cyanine dye were tested by using the same TLSC to match the ${J}_{SC}$ with the integrated $\,{J}_{SC}$, and a matte black background was placed on the back of the tested TLSC to eliminate the illumination from the environment or reflection (double pass) for both *PCE* and *EQE* measurements. We also utilize the same PV cells mounted around the edge to eliminate any PV-to-PV variations in performance.

### Optical Modeling

The reabsorption and forward emission losses were estimated with luminophore (Cy7-CA) absorption, emission spectra, distance *d* and TLSC plate length *L*. The TLSC system optical efficiencies, in considering reabsorption losses from the overlap in the absolute absorption and normalized emission spectra were numerically evaluated in Matlab as a function of distance *d*, plate length *L*, plate thickness ${t}_{0}$ and dye/polymer film thickness *t*. The complete equations used in the simulations are provided in the Supplemental Information.

### Electronic Structure Calculations

The geometries of Cy7-CA, Cy7-NEt_2_-I, and Cy7.5-NEt_2_-I were optimized in their ground and first excited electronic states to elucidate the relaxation motions responsible for the enhanced Stokes shifts of Cy7-NEt_2_-I and Cy7.5-NEt_2_-I. The charged side chain on the Cy7-CA and two ethyl groups on the two nitrogen atoms terminating the polymethine backbone of Cy7.5-NEt_2_-I were replaced by methyl groups to reduce the cost of calculations. This is expected to have little effect on the Stokes shifts because the HOMO and LUMO do not extend to these side chains (as shown in Fig. 2). Calculations were performed at the linear response time-dependent density functional level of theory using the TeraChem software package^46–49^. The CAM-B3LYP functional^50^ and 6–31 G* basis was used, and all calculations were performed in the gas phase. Though sometimes predicting inaccurate vertical excitation energies, TDDFT is known to give an accurate description of the shape of the excited state potential energy surface (e.g. SS)^51^. These calculations were enabled by the Extreme Science and Engineering Discovery Environment (XSEDE)^52^. Torsion angles are defined as the mean of the two C1-C2-N-C3 dihedral angles where C2 is the central carbon atom of the polymethine chain and N is the nitrogen of the amino group.

## Electronic supplementary material

Supplementary Information
