# Supplementary material for: Impact of Stokes Shift on the Performance of Near-Infrared Harvesting Transparent Luminescent Solar Concentrators
Source: Sci Rep. 2018 Nov 5;8:16359. doi: 10.1038/s41598-018-34442-3 (PMC6218549; doi:10.1038/s41598-018-34442-3)
Supplement: Supplementary file 1 — Supplementary Information [file 41598_2018_34442_MOESM1_ESM.docx]

Supporting Information for

**Impact of Stokes Shift on the Performance of Near-Infrared Harvesting Transparent Luminescent Solar Concentrators**

Chenchen Yang,^1)^ Jun Zhang^2)^, Wei-Tao Peng^2)^, Wei Sheng^2)^, Dianyi Liu^1)^, Padmanaban S. Kuttipillai^1)^, Margaret Young^1)^ Matthew R. Donahue^1)^, Benjamin G. Levine^2)^, Babak Borhan^2)^, Richard R. Lunt^1), 3) a)^

1) Department of Chemical Engineering and Materials Science, Michigan State University, East Lansing, MI, 48824 USA

2) Department of Chemistry, Michigan State University, East Lansing, MI, 48824 USA

3) Department of Physics and Astronomy, Michigan State University, East Lansing, MI, 48824 USA

a) [rlunt@msu.edu](mailto:rlunt@msu.edu)

**SI-1 Cyanine Dyes Synthesis and General Instrumentation:**

**1) Cy7-CA:**

**1-(5-carboxypentyl)-3,3-dimethyl-2-((*E*)-2-((*E*)-3((*E*)-2-(1,3,3-trimethylindolin-2ylidene)ethylidene)cyclohex-1-enyl)vinyl)-3*H*-indolium chloride** (Cyanine7 carboxylic acid or Cy7-CA) (Lumiprobe) was characterized as received without further purification.

**2) Cy7-NEt_2_-I and Cy7.5-NEt_2_-I:**

**Materials and General Instrumentations:**

Solvents and reagents used for reactions were purchased from commercial sources.^1^ HPLC grade acetonitrile (Sigma-Aldrich) was used for all reactions. Column chromatography was performed using SiliCycle silica gel (230-400 mesh). Thin layer chromatography (TLC) with fluorescent indicator was purchased from Analtech. ^1^H-NMR and ^13^C-NMR spectra were obtained on Varian Inova 500 MHz instruments and were reported in parts per million (ppm) relative to the solvent resonances (*δ*), with coupling constants ($J_{NMR}$) in Hertz (Hz). HRMS analysis was performed on a Q-TOF Ultima system using electrospray ionization in positive mode. UV-Vis spectra were recorded on an Agilent Cary 100 series spectrophotometer. PL was recorded on an Fluorolog equipped with ISA instrument (Horiba, Jobin-Yvon).

**General synthesis:**


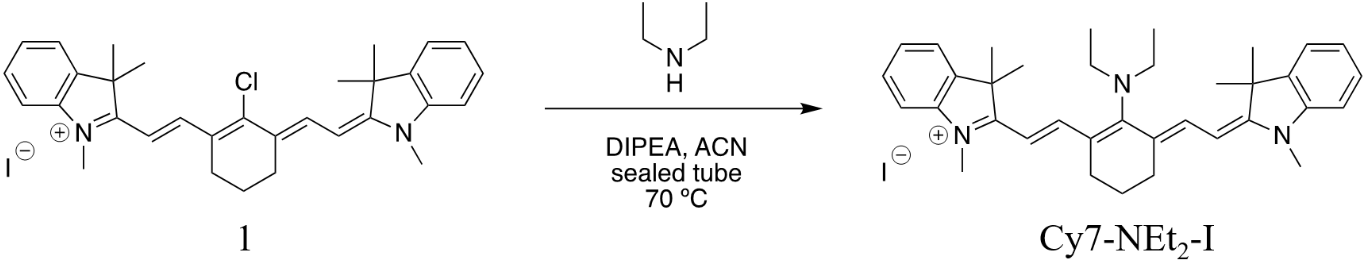


**2-((*E*)-2-((*E*)-2-(diethylamino)-3-(2-((*E*)-1,3,3-trimethylindolin-2-ylidene)ethylidene)cyclohex-1-en-1-yl)vinyl)-1,3,3-trimethyl-3*H*-indol-1-ium iodide (Cy7-NEt_2_-I)**

Compound **1** is synthesized according to the previous reported procedures.^1^ To a solution of **1** (74 mg, 0.10 mmol), diisopropylethylamine (DIPEA) (35 μL, 2 equiv, 0.20 mmol) in acetonitrile (2 mL) was added freshly distilled diethylamine (206 μL, 20 equiv, 2.0 mmol ) under nitrogen. The mixture was stirred at 70 °C and the reaction was monitored by LC-MS. Upon completion, typically in 48 h, the mixture was concentrated on reduced pressure and purified by flash column (100% DCM gradually to 5% MeOH/DCM). The product was isolated as a dark solid (21 mg, 0.032 mmol, 32 %). **^1^H NMR** (500 MHz, Chloroform-d) *δ* 7.52 (*d*, $J_{NMR}$ = 13.6 Hz, 2H), 7.34 – 7.26 (m, 4H), 7.11 (m, 2H), 7.02 (*d*, $J_{NMR}$ = 7.9 Hz, 2H), 5.80 (*d*, $J_{NMR}$ = 13.6 Hz, 2H), 3.65 (*q*, $J_{NMR}$ = 6.9 Hz, 4H), 3.52 (s, 6H), 2.49 (*t*, $J_{NMR}$ = 6.5 Hz, 4H), 1.82 (*p*, $J_{NMR}$ = 6.5 Hz, 2H), 1.60 (s, 12H), 1.28 (*t*, $J_{NMR}$ = 6.9 Hz, 6H). **^13^C NMR** (126 MHz, Chloroform-d) *δ* 174.15, 169.83, 143.19, 142.52, 140.06, 128.57, 125.76, 123.76, 121.97, 109.50, 96.95, 49.47, 48.03, 31.36, 29.07, 24.88, 21.98, 14.84. **HRMS (ESI+)**: calcd for C_36_H_46_N_3_^+^ [M]^+^ 520.3690, found 520.3692.


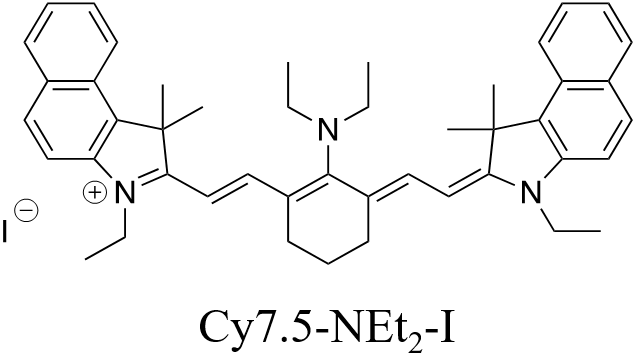


**2-((*E*)-2-((*E*)-2-(diethylamino)-3-((*E*)-2-(3-ethyl-1,1-dimethyl-1,3-dihydro-2*H*-benzo[*e*]indol-2-ylidene)ethylidene)cyclohex-1-en-1-yl)vinyl)-3-ethyl-1,1-dimethyl-1*H*-benzo[*e*]indol-3-ium iodide (Cy7.5-NEt_2_-I)**

**Cy7.5-NEt_2_-I** was prepared according to the same procedure described for **Cy7-NEt_2_-I**. The product was isolated as a dark solid (36 mg, 0.046 mmol, 46 %). **H NMR** (500 MHz, Chloroform-*d*) δ 8.07 (*d*, $J_{NMR}$ = 8.5 Hz, 2H), 7.89 (*d*, $J_{NMR}$ = 8.9 Hz, 4H), 7.69 (*d*, $J_{NMR}$ = 13.7 Hz, 2H), 7.58 – 7.51 (m, 2H), 7.42 – 7.37 (m, 2H), 7.34 (*d*, $J_{NMR}$ = 8.8 Hz, 2H), 5.89 (*d*, $J_{NMR}$ = 13.7 Hz, 2H), 4.15 (*q*, $J_{NMR}$ = 7.2 Hz, 4H), 3.70 (*q*, $J_{NMR}$ = 6.9 Hz, 4H), 2.53 (*t*, $J_{NMR}$ = 6.4 Hz, 4H), 1.93 (s, 12H), 1.89 – 1.85 (m, 2H), 1.44 (*t*, $J_{NMR}$ = 7.2 Hz, 6H), 1.35 (*t*, $J_{NMR}$ = 6.9 Hz, 6H). **^13^C NMR** (126 MHz, Chloroform-*d*) *δ* 173.00, 170.56, 142.05, 139.64, 132.46, 131.29, 130.56, 130.10, 128.33, 127.57, 125.62, 124.37, 121.82, 110.20, 96.22, 50.06, 49.39, 39.18, 28.59, 28.44, 25.01, 22.05, 14.96, 12.31. **HRMS (ESI+)**: calcd for C_46_H_54_N_3_^+^ [M]^+^ 648.4319, found 648.4318.


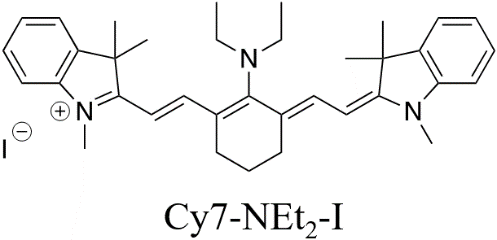

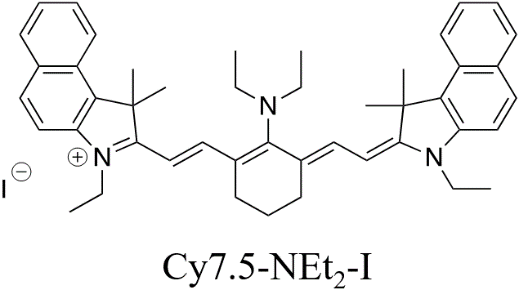

**SI-2 *J-V* and *EQE* Schematics and EQEs of three TLSCs and Table:**


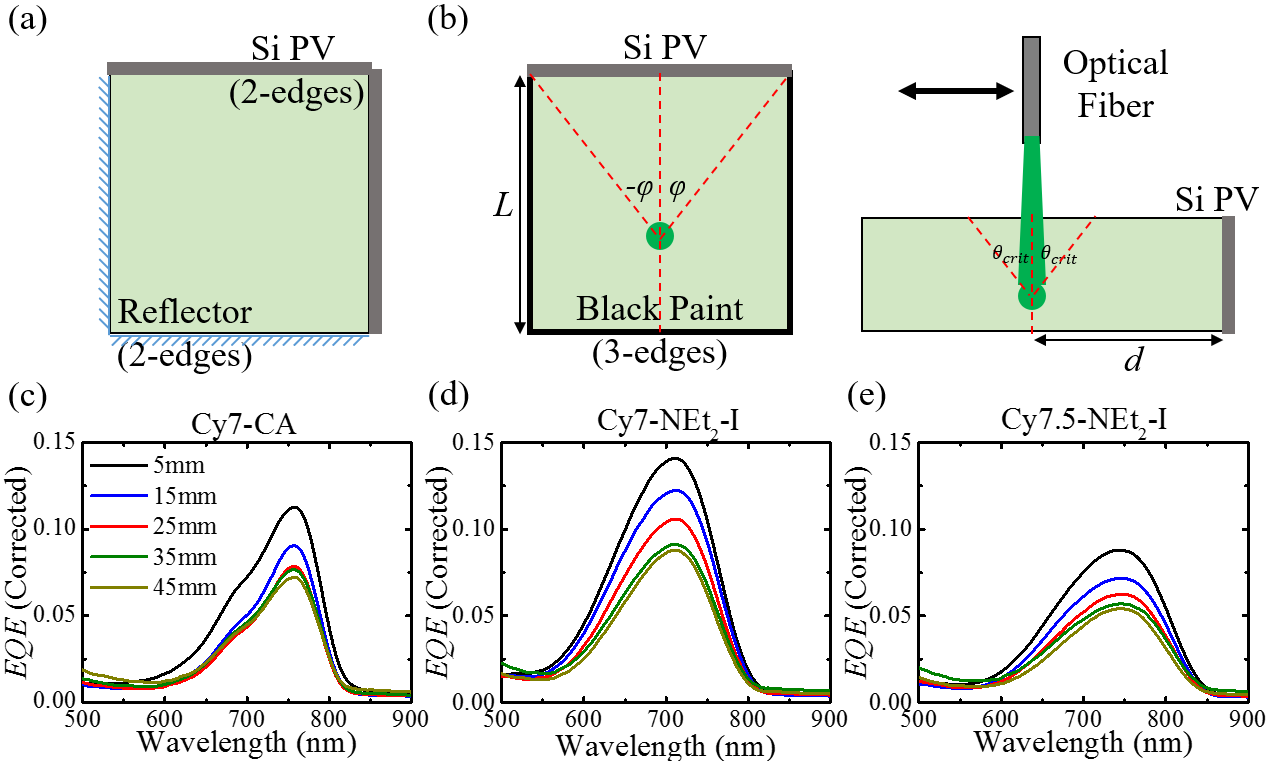
 Figure S1

| *Int. Jsc*  (mA/cm^2^) | 5mm | 15mm | 25mm | 35mm | 45mm |
| --- | --- | --- | --- | --- | --- |
| Cy7-CA | 1.17 | 0.95 | 0.92 | 0.88 | 0.87 |
| Cy7-NEt_2_-I | 1.55 | 1.36 | 1.16 | 1.07 | 0.96 |
| Cy7.5-NEt_2_-I | 1.05 | 0.86 | 0.76 | 0.75 | 0.66 |

Table S1

**SI-3 *T*+*R*+*EQE* ≤ 1 Check:**


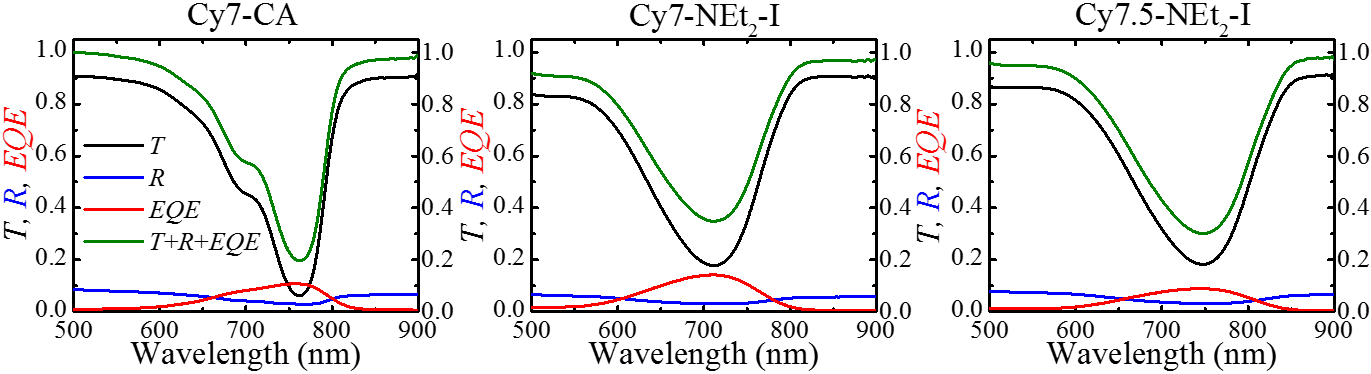


Figure S2

**SI-4 Numerical Modeling:**

The LSC system efficiency can be calculated from the Equation (S1) below:^2,3^

$\eta_{LSC}=\eta_{Opt}^{*}\cdot\eta_{PV}^{*}=\left( 1-R_{f} \right)\cdot\eta_{Abs}\cdot\eta_{PL}\cdot\eta_{Trap}\cdot\eta_{RA}\cdot\eta_{PV}^{*} (S1)$

where $R_{f}=\left( n_{sub}-1 \right)^{2}/\left( n_{sub}+1 \right)^{2}$, $\eta_{Trap}=\sqrt{1-1/n_{sub}^{2}}$, $\eta_{Abs}=\int_{300nm}^{E_{g}^{lum}} AM1.5G(\lambda)\cdot A\left( \lambda\right)d\lambda/\int_{300nm}^{\infty} AM1.5G(\lambda)d\lambda$, and $A\left( \lambda\right)$ is the single-path absolute absorption spectrum of the luminophore/polymer composite film, $E_{g}^{lum}$ is the bandgap of the luminophore and $AM1.5G(\lambda)$ is the air mass 1.5 global solar photon flux spectrum. The PV efficiency under the downconverted luminescence is: $\eta_{PV}^{*}=\left( \frac{\eta_{PV}\left( AM1.5G \right)}{\eta_{Abs}^{PV}\left( AM1.5G \right)} \right)\cdot\frac{\int{EQE}_{PV}\left( \lambda^{'} \right)\cdot PL(\lambda^{'})d\lambda^{'}}{\int PL(\lambda^{'})d\lambda^{'}}$, where $\eta_{EQE}\left( \lambda^{'} \right)$ is the external quantum efficiency of the edge-mounted PV as a function of wavelength, $PL(\lambda^{'})$ is the luminophore photoluminescence emission spectrum in and $\eta_{Abs}^{PV}\left( AM1.5G \right)$ is the absorption efficiency of the PV material rather than the luminophore which has been defined above. $\eta_{PL}$ is the measured photoluminescence quantum yield of the luminophore in luminophore/polymer composite film:

$$\eta_{RA}=\frac{1-\eta_{RAP}}{1-\eta_{RAP}\cdot\eta_{PL}\cdot\eta_{Trap}} (S2)$$

where the reabsorption probability, $\eta_{RAP}$ is integrated over all emission angles and the absorptive path length is corrected for each take-off angle in a rectilinear system as:^4,5^

$\eta_{RAP}=\frac{\int_{0}^{\infty} d\lambda\int_{\theta_{crit}}^{\pi/2} d\theta\int_{-\varphi}^{\varphi} sin(\theta)\cdot PL(\lambda)\cdot\left( 1-exp\left[ -A\left( \lambda\right) \cdot\frac{\frac{d}{sin(\theta)\cdot cos(\theta)} \cdot\frac{t}{{(t}_{0}+t)}}{t} \right] \right)d\varphi}{\int_{0}^{\infty} d\lambda\int_{\theta_{crit}}^{\pi/2} d\theta\int_{-\varphi}^{\varphi} sin(\theta)\cdot PL(\lambda)d\varphi} (S3)$

where the critical angle (emission cone) is $\theta_{crit}={sin}^{-1}(1/n_{sub}),$ $\theta$ is the azimuth relative to the normal of the LSC waveguide, $t$ is the thickness of the luminophore/polymer composite film, $t_{0}$ is the waveguide thickness, and $\varphi={arctan}^{-1}(2L/d)$ is the in-plane rotation angle, *L* is the plate length and *d* is the distance from excitation source to edge-mounted Si PV (as shown in Figure S1b). We note that Equation 2 in the main text can also be obtained, and is equivalent to, starting from $\eta_{LSC}=J_{SC}{\cdot V}_{OC}\cdot FF/P_{0}$ by substituting the equation for $J_{SC}=q\int{EQE}_{LSC}\left( \lambda\right)AM1.5G\left( \lambda\right)d\lambda$ with ${EQE}_{LSC}\left( \lambda\right)$ defined in Eq. 1, the definition of the incident power ($P_{0}$) as the integrated solar spectrum, and recognizing that $\eta_{PV}^{*}$ represents the $FF$ and $V_{OC}$ components from the PV cell under illumination of the luminophore.

**Reference:**

1. Samanta, A., Vendrell, M., Das, R. & Chang, Y.-T. Development of photostable near-infrared cyanine dyes. *Chem. Commun.* **46,** 7406–7408 (2010).

2. Debije, M. G. & Verbunt, P. P. C. Thirty Years of Luminescent Solar Concentrator Research: Solar Energy for the Built Environment. *Adv. Energy Mater.* **2,** 12–35 (2012).

3. Yang, C. & Lunt, R. R. Limits of Visibly Transparent Luminescent Solar Concentrators. *Adv. Opt. Mater.* **5,** 1600851 (2017).

4. Goetzberger, A. & Greube, W. Solar energy conversion with fluorescent collectors. *Appl. Phys.* **14,** 123–139 (1977).

5. Batchelder, J. S., Zewail, A. H. & Cole, T. Luminescent solar concentrators. 2: Experimental and theoretical analysis of their possible efficiencies. *Appl. Opt.* **20,** 3733–3754 (1981).
